# Sustainable Plastics with High Performance and Convenient Processibility

**DOI:** 10.1002/advs.202405301

**Published:** 2024-07-19

**Authors:** Guogang Xu, Lei Hou, Peiyi Wu

**Affiliations:** ^1^ State Key Laboratory for Modification of Chemical Fibers and Polymer Materials, College of Chemistry and Chemical Engineering Donghua University Shanghai 201620 China

**Keywords:** 3D printing, hydrogen bond, mechanical robustness, sustainable polymer, water‐processable plastic

## Abstract

Designing and making sustainable plastics is especially urgent to reduce their ecological and environmental impacts. However, it remains challenging to construct plastics with simultaneous high sustainability and outstanding comprehensive performance. Here, a composite strategy of in situ polymerizing a petroleum‐based monomer with the presence of an industrialized bio‐derived polymer in a quasi‐solvent‐free system is introduced, affording the plastic with excellent mechanical robustness, impressive thermal and solvent stability, as well as low energy, consumes during production, processing, and recycling. Particularly, the plastic can be easily processed into diverse shapes through 3D printing, injection molding, etc. during polymerization and further reprocessed into other complex structures via eco‐friendly hydrosetting. In addition, the plastic is mechanically robust with Young's modulus of up to 3.7 GPa and tensile breaking strength of up to 150.2 MPa, superior to many commercially available plastics and other sustainable plastics. It is revealed that hierarchical hydrogen bonds in plastic predominate the well‐balanced sustainability and performance. This work provides a new path for fabricating high‐performance sustainable plastic toward practical applications, contributing to the circular economy.

## Graphical abstract

Sustainable plastic is achieved with excellent mechanical robustness, impressive thermal and solvent stability as well as low energy consumption during production, processing, and recycling. The plastic can be easily processed into diverse shapes via 3D printing, injection molding, hydrosetting, etc. Meanwhile, the plastic shows *E* up to 3.7 GPa and *σ*_b_ up to 150.2 MPa, superior to many commercially available plastics and other sustainable plastics.

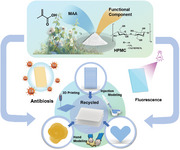

## 1 Introduction

Plastics are indispensable in almost every aspect of our daily life owing to their lightweight, excellent durability, and tunable mechanical performance, which leads to the boomed manufacture of plastic products amounting to 400 million tonnes per year over the world.^[^ ^1^ ^]^ Nevertheless, the massive production and disposal of plastics have brought about accumulation of plastic waste in the natural environment, from land to ocean, resulting in the squander of carbon resources as well as increased ecological and environmental concerns.^[^ ^2^ ^]^ From the perspective of material design, it is urgent to develop sustainable plastics to reduce their negative impacts. Efforts to address such an issue include producing plastics from biomass and bioproducts,^[^ ^3^ ^]^ developing degradable plastics,^[^ ^4^ ^]^ and recycling plastic waste mechanically or chemically.^[^ ^5^ ^]^ Sustainability not only refers to reducing the amount of plastic waste that contaminates the environment but also decreasing the share of CO_2_ emissions during the manufacturing, reuse, and recycling of plastics.^[^ ^6^ ^]^ Unfortunately, it is difficult for current strategies to satisfactorily meet all the goals. For example, degradable polymers,^[^ ^7^ ^]^ in most situations, undergo inefficient degradation and consequently, give rise to new environmental problems such as microplastic pollution. Additionally, many available techniques of recycling plastic waste are energy‐intensive.^[^ ^8^ ^]^ Developing sustainable plastics means to tackle the problem along the full life cycle of plastics, including feedstock, synthesis, processing, and end‐of‐life management. Thus, it would be of great significance to construct sustainable plastic from a whole‐life perspective.

In addition to sustainability, the overall performance of the plastic, such as mechanical properties, and thermal and wet stability, plays a predominant role in practical applications, contributing to the irreplaceability of plastics in the modern society.^[^ ^9^ ^]^ Currently, many sustainable plastics come at the expense of material properties. For instance, bioplastics, made from various biomass feedstocks, can efficiently reduce the reliance on non‐renewable fossil fuels and diminish carbon footprint. However, they frequently face the risk of wet instability due to water‐induced breaking down of hydrogen bonds among bio‐based polymer chains.^[^ ^10^ ^]^ For plastics with sustainable processing, which implies fabricating polymers^[^ ^11^ ^]^ into desired structures with reduced carbon emissions, they mostly exhibit limited mechanical performance owing to fragile intra‐/inter‐ chain interactions. From these points of view, molecular interaction is critical in determining the comprehensive properties of plastics, suggesting a potential method of delicately manipulating molecular interactions to fabricate plastics with well‐balanced sustainability and performance.

**Figure 1:**
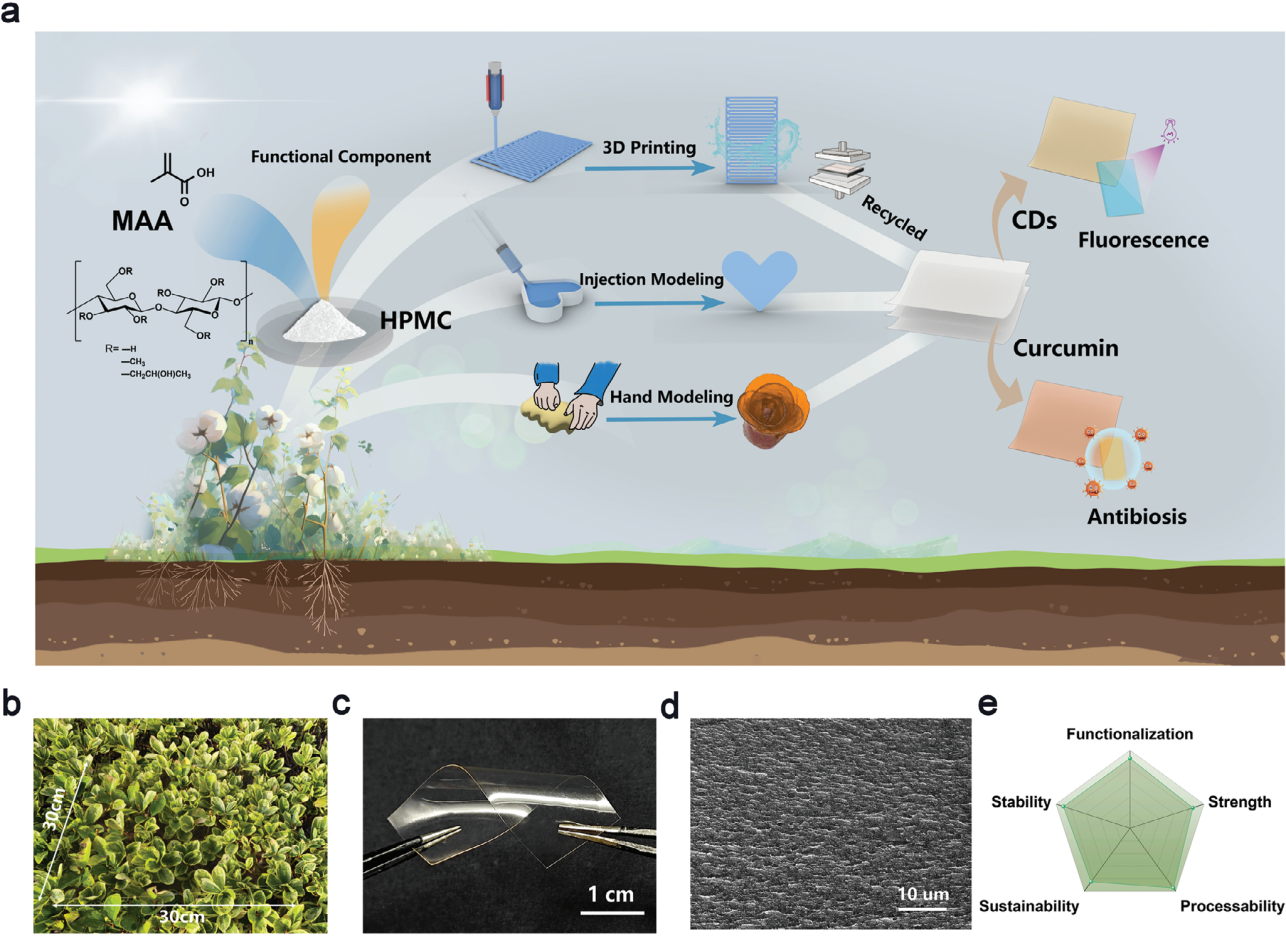
Fabrication of the sustainable plastic: a) Schematic illustration of the preparation and processing of the HPMC/PMAA plastic; Photograph demonstrating of the large area preparation b) and flexibility c) of the plastic; d) SEM image of the cross section of the plastic; e) Advantages of the plastics.

Here, we present a composite strategy by in situ polymerizing a petroleum‐based monomer in the presence of an industrialized bio‐derived polymer to construct high‐performance plastics with low energy consumption during production, processing, and recycling (**Figure** **1**a). Particularly, employing a liquid monomer as the solvent and a small amount of water as the co‐solvent, the rheological property of the polymerization precursor can be conveniently regulated, facilizing the sustainable processing of plastic into diverse shapes via 3D printing, injection molding, hand molding, etc. Afterward, the plastics can be reprocessed into other configurations through an eco‐friendly hydrosetting method. In addition, recycling of the plastic could be achieved at mild conditions owing to its supramolecular nature. Meanwhile, the plastic exhibits excellent mechanical robustness, and impressive thermal and water stability by virtue of hierarchical hydrogen bonding interactions, demonstrating a unique balance between sustainability and performance. This work provides an effective strategy to develop sustainable plastics with outstanding overall performance toward actual applications.

## 2 Results and Discussion

### 2.1 Fabrication of the Sustainable Plastic

Hydroxypropyl methyl cellulose (HPMC) is a hydrophilic polymer derived from cellulose, one of the most abundant biopolymers on earth, and has long been commercialized and widely used in foods, pharmaceuticals, cosmetics, and buildings, demonstrating a suitable feedstock to produce sustainable plastics. Herein, the sustainable plastic is prepared via in situ polymerization of a liquid monomer, methyl acrylic acid (MAA) with the presence of HPMC in a quasi‐solvent‐free precursor, where only a small amount of water is needed for tuning the hydrogen bonding structures and rheological properties. Physically mixing HPMC powders with the monomer, MAA, at room temperature affords a homogeneous viscous liquid (Figure S1, Supporting Information), whose viscosity and rheological property could be conveniently adjusted by varying the HPMC/MAA ratio or adding a small amount of water (Figures S2 and S3, Supporting Information). In such a process, the key point lies in the fact that HPMC is partially compatible with MAA, that is, HPMC can be swollen by MAA but can not dissolve in MAA, as displayed in Figure S4 (Supporting Information). Hydrogen bonding between MAA and HPMC plays an important role in such a swelling process. Low‐field‐^1^H nuclear magnetic resonance (LF‐^1^H NMR) spectroscopy reflects the freedom degree of protons by measuring the proton spin‐spin relaxation time, where a longer relaxation time indicates more free protons. In Figure S5 (Supporting Information), the *T*_2_ values related to MAA and HPMC all shift to lower ones after mixing, indicating the protons are more confined in the mixture. Both HPMC and MAA are compatible with water, as revealed by the decreased *T*_2_ values after adding a proper amount of water into the HPMC/MAA mixture, making it convenient to tune the viscosity of the reaction system for diverse processing methods.

After polymerization, a transparent plastic can be obtained (Figure 1b; Figure S6, Supporting Information). It is flexible enough to be twisted without wrinkling or cracking (Figure 1c). Though the reaction mixture is viscous, the polymerization of MAA is hardly affected, which could be confirmed by the obviously reduced intensity of the carbon–carbon double bond in time‐dependent FTIR spectra during polymerization (Figure S7, Supporting Information). In addition, the SEM image (Figure 1d) indicates that the HPMC/PMAA plastic holds a homogeneous and dense structure, confirming the uniform mixing of MAA and HPMC.

Compared with our previous work,^[^ ^12^ ^]^ where robust hydroplastics were constructed from polymer complexes, notable benefits with this approach to fabricate sustainable plastic are (Figure 1e): 1) full utilization of feedstocks can be achieved; 2) the precursor mixture of proper viscosity can be easily processed into diverse shapes through different methods, including 3D printing, injection molding, and compression molding; 3) no complicated purification or post‐treatment is required except for air‐drying; 4) incorporating functional components into the plastic is highly available. All of these would contribute greatly to the reduction of carbon emissions during plastic manufacturing.

### 2.2 Plastic Properties

**Figure 2:**
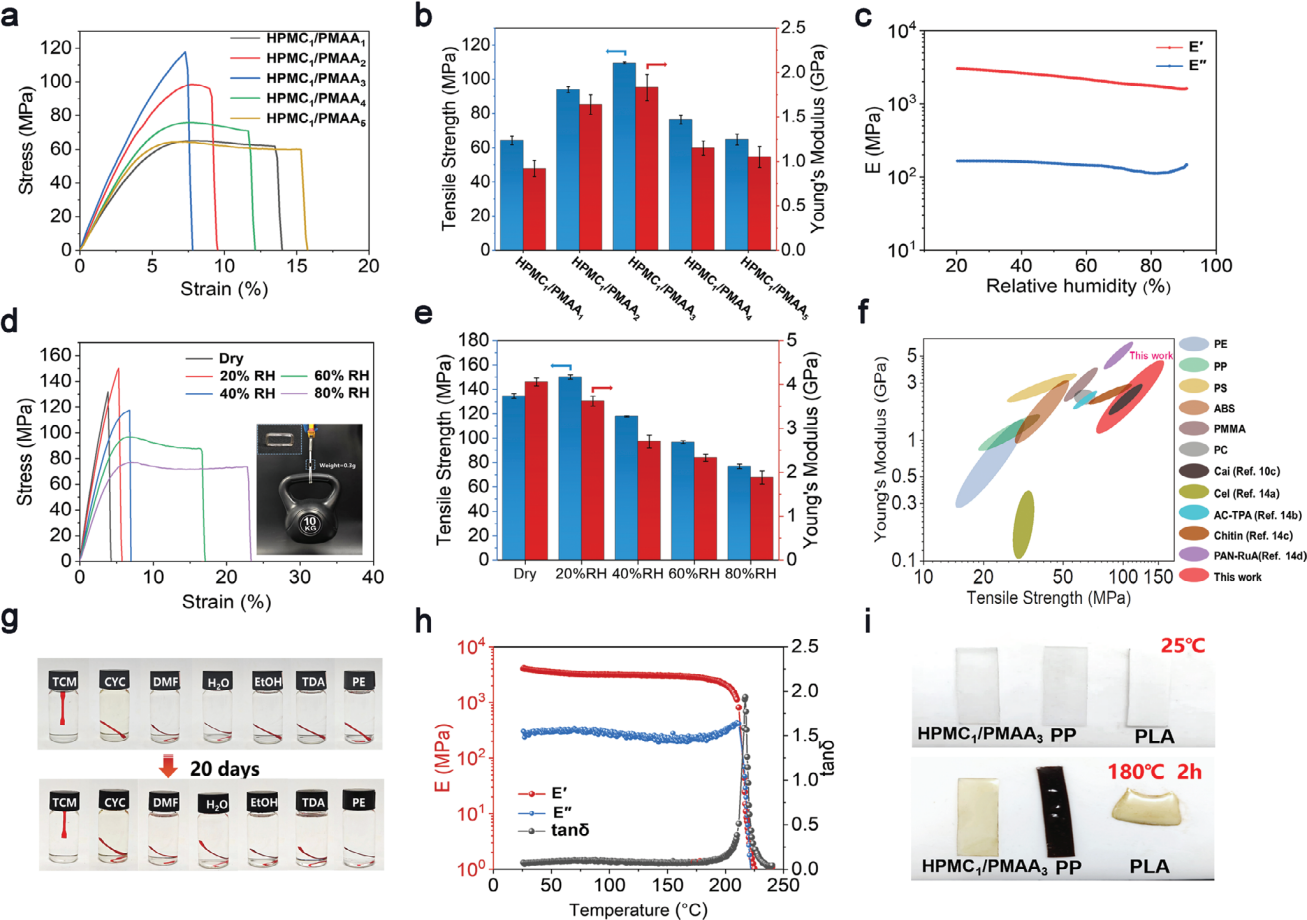
Plastic Properties: Tensile stress–strain curves a) and corresponding breaking strength and Young's modulus of (b) HPMC/PMAA plastics with different compositions equilibrated under 50% RH; c) DMA curves of humidity sweep of HPMC_1_/PMAA_3_ plastic; Tensile stress–strain curves (The insert shows the load‐bearing capacity) d) and corresponding breaking strength and Young's modulus e) of HPMC_1_/PMAA_3_ plastic under different RH; f) Comparison of mechanical properties of HPMC/PMAA plastics with commercial plastics^[^ ^13^ ^]^ and other sustainable plastics^[^ ^10^, ^14^ ^]^; g) Photographs of the HPMC_1_/PMAA_3_ plastic stored in different solvents for 20 days (Note that the plastics in the images are dyed with Disperse Red 1 for better demonstration); h) DMA curves of temperature sweep of HPMC_1_/PMAA_3_ plastic; i) Photograph of the thermal stability comparison of HPMC_1_/PMAA_3_ plastic with polypropylene (PP) and polylactic acid (PLA).

Uniaxial tensile testing was employed to investigate the mechanical properties of HPMC_1_/PMAA_m_ plastics, where “m” represents MAA/HPMC weight ratios in the polymerization precursors. As displayed in **Figure** **2**a,b, the plastic is mechanically strong and robust with Young's modulus (*E*) ranging from 0.9 to 1.8 GPa, and tensile breaking strength (*σ*_b_) of 64.3 to 109.5 MPa. Herein, the HPMC_1_/PMAA_3_ plastic demonstrates optimized *E* and *σ*_b_, which might be related to the well‐balanced hydrogen bonds in this formula.^[^ ^12^ ^]^ Dynamic Mechanical Analysis (DMA) was performed to examine the wet resistance of the plastic (Figure 2c). During a humidity sweep from 20 to 80% relative humidity (RH), the elastic modulus of HPMC_1_/PMAA_3_ plastic exhibits only a slight decrease and is located at 1.9 GPa even at 80% RH, demonstrating excellent moisture stability of the plastic. Further tensile tests show that the *σ*_b_ remains higher than 75 MPa even at 80% RH (Figure 2d,e). It is noted that the plastic is brittle after complete drying and becomes tough at the intermedium RH, suggesting that a proper amount of water would toughen the plastic (Figure S8, Supporting Information). Specifically, a 0.3 g HPMC_1_/PMAA_3_ rectangular connector prepared by 3D printing can bear a weight of 10 kg without failure (Figure 2d) at an ambient environment (room temperature and 40–50% RH). The mechanical performance of HPMC/PMAA is superior to many commercially available plastics and other sustainable plastics (Figure 2f).

Although HPMC and PMAA are both hydrophilic, the HPMC/PMAA plastic exhibits limited swelling with neglected shape change after immersing in water for 20 days, demonstrating its stability in an aqueous environment (Figure S9, Supporting Information). For the HPMC_1_/PMAA_3_ plastic, the water content at swelling equilibrium is ≈38%. Meanwhile, the *E* and *σ*_b_ reduce to 123.3 MPa and 19.4 MPa, respectively, owing to the plasticization of water molecules on the polymer chains (Figure S10, Supporting Information). This behavior, to some extent, provides the plastic with hydroplastic processing, where the shape of the plastic could be molded in water and subsequently fixed by drying. Additionally, the plastic remains intact after incubation in organic solvents, indicating its stability in organic environments (Figure 2g; Figure S11, Supporting Information).

Benefited from the synergistic effect of rigid HPMC components and dense hydrogen bonds, the HPMC/PMAA plastics exhibit excellent thermal stability with glass transitions higher than 200 °C (Figure 2h; Figure S12, Supporting Information), superior to many commonly used plastics (≈70 °C for PET, ≈85 °C for PVC, ≈100 °C for PS, 90≈120 °C for ABS, etc.) In the DMA curves, the elastic modulus remains nearly unchanged in the temperature range of 25 to 180 °C. In addition, it is directly observed that, after incubation at 180 °C for 2 h, the HPMC_1_/PMAA_3_ plastic could maintain a stable shape, whereas some other plastics have already softened and deformed (Figure 2i).

### 2.3 Sustainable Processing and Recycling of the Plastic

**Figure 3:**
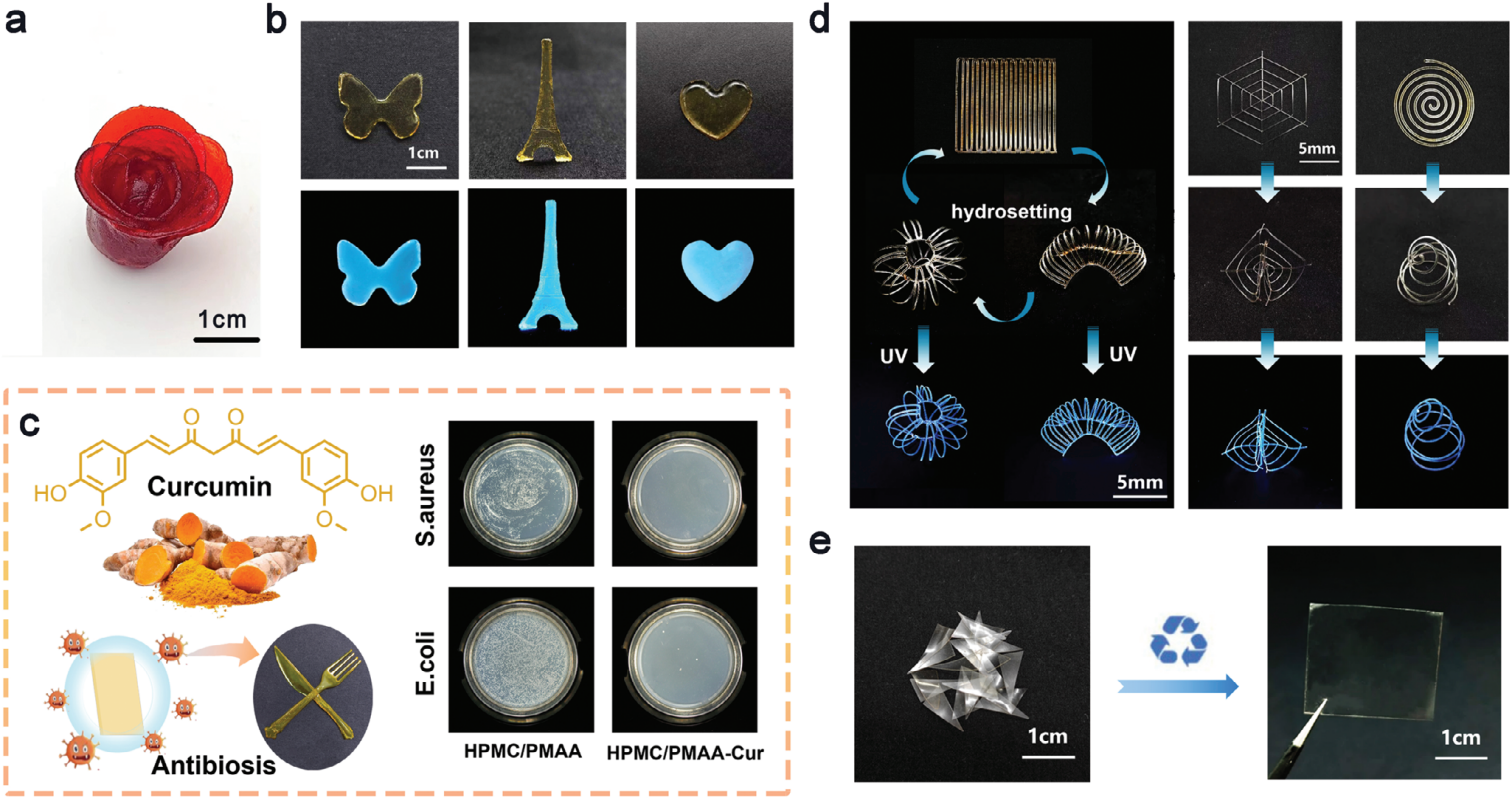
Sustainable processing and recycling of the HPMC/PMAA plastic: a) Photographs of the “flower” plastic is shaped by hand modeling; b) Injection molding of the plastic functionalized with carbon dots (CDs) into different shapes; c) Injection molding of the plastic functionalized with curcumin into tableware with antibacterial properties; d) Fine structures produced by 3D printing and hydrosetting of the plastic functionalized with CDs; e) Photographs of plastic recycling.

In the traditional processing of plastic products, polymers need to be heated well above their glass transition temperature or flow temperature, which is energy intensive. In addition, side effects, including oxidation of vulnerable functional groups and incorporation of plasticizers, exist during high‐temperature processing. In this work, the rheological property of the polymerization precursor can be conveniently regulated, facilitating the sustainable molding process with different methods, such as hand molding, injection molding, 3D printing, etc. After polymerization, plastics with diverse shapes can be obtained. As shown in **Figure** **3**a, the plastic can be shaped into a “flower” by hand modeling. In Figure 3b, the “butterfly”, “tower” and “heart” shaped plastics are generated with injection molding at room temperature. Herein, it is highly available to introduce other components into the polymerization precursor through physical blending, endowing the final plastic with various functionalities.^[^ ^15^ ^]^ For example, carbon dots (CDs) can be incorporated into the polymerization precursor,^[^ ^16^ ^]^ making the plastic emit blue fluorescence under the ultraviolet lamp (Figure 3b). Besides, curcumin can be loaded into plastic to furnish it with antibacterial properties^[^ ^17^ ^]^ (Figure 3c). Furthermore, plastics with delicate structures can be achieved via 3D printing^[^ ^18^ ^]^ (Figure 3d). Owing to water‐induced softening, the HPMC/PMAA plastic can be easily reprocessed into complicated shapes using a facile and sustainable hydrosetting method, which contains 1) programming the plastic into desired shapes after swelling in water and 2) air‐drying the wet plastic in ambient environments.^[^ ^19^ ^]^ Meanwhile, the hydrosetting process is quite reversible (Figure 3d and Figure S13, Supporting Information).

In addition to sustainable processing, the HPMC/PMAA plastic demonstrates good recyclability. The plastic exhibits a supramolecular nature and is dynamically cross‐linked by hydrogen bonds, making it recyclable under mild conditions. After immersing in water to equilibrium, the plastic fragments can be hot‐pressed into a uniform film at 70 °C, 5 MPa for 12 h, as displayed in Figure 3e. Herein, the mechanical performance is almost maintained after reconstruction (Figure S14, Supporting Information).

### 2.4 Molecular Basis for the High‐Performance Sustainable Plastic

**Figure 4:**
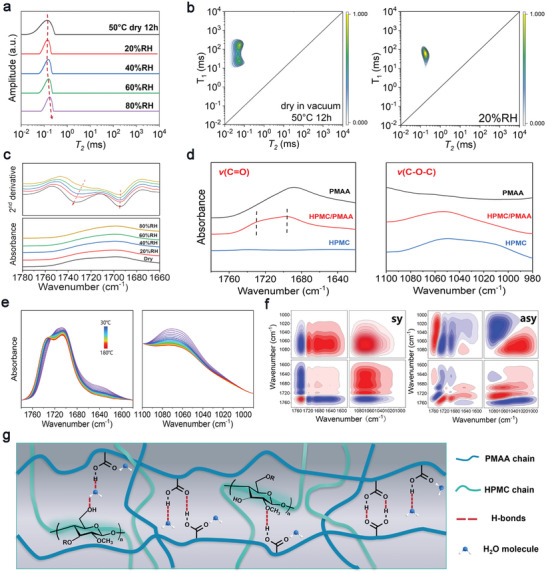
Molecular basis for the high‐performance sustainable HPMC_1_/PMAA_3_ plastic: a) LF‐^1^H NMR spectra of the plastic equilibrated under different RH; b) 2D LF‐^1^H NMR spectra of the plastic in different states; c) FTIR spectra of the plastic in the 1780‐1660 region equilibrated under different RH; d) FTIR spectra of HPMC, PMAA and HPMC/PMAA in dry states of the *v*(C═O) and *v*(C─O─C) regions; e) Temperature‐dependent FTIR spectra of the plastic from 30 to 180 °C in the *v*(C═O) and *v*(C─O─C) regions; f) 2Dcos synchronous and asynchronous spectra generated from (e). In 2Dcos spectra, the warm colors (red) represent positive intensities, while cold colors (blue) represent negative ones; g) Schematic diagram of internal interactions in the plastic.

Hydrogen bonds play a crucial role in excellent mechanical performance, water, and thermal resistance as well as sustainable processing and recycling. First, LF‐^1^H NMR spectra were carried out to examine the water species in the HPMC_1_/PMAA_3_ plastic. After incubation in different RH to an equilibrium, the *T*_2_ values of all protons in the plastic stay below 1 ms (**Figure** **4**a,b and Figure S15, Supporting Information), indicating that protons in absorbed water molecules are strongly bonded with polymer chains and even hold similar degree of freedom with covalently bonded protons. It is noticed that *T*_2_ peaks exhibit a narrowing behavior after moisture absorption. Meanwhile, the association of peaks in the 2D LF‐^1^H NMR spectra with the presence of water could be observed. Such observations could be closely related to the hydrogen bonds between polymer chains and water molecules, where water molecules could participate in the bridge of hydrogen bonds to crosslink the polymer chains. The strongly bonded water molecules in the plastic could be also confirmed from the TGA curves (Figures S16 and S17,Supporting Information), where the weight loss related to water evaporation continues until 200 °C. FTIR spectroscopy is further conducted to investigate the hydrogen bonding evolution after absorbing water. As displayed in Figure S18 (Supporting Information) the absorbed water can be observed by the increased intensity at ≈3400 cm^−1^, which is assigned to the stretching vibration of the OH group. Through careful inspection, it could be noticed that the band related to *v*(C═O) of PMAA exhibits apparent changes with the presence of water (Figure 4c).

To be more specific, two bands, located at 1695 and 1730 cm^−1^, have been recognized from the dry HPMC_1_/PMAA_3_ plastic, which can be assigned to *v*(C═O) with strong and weak hydrogen bonds, respectively. After moisture absorption, the band at 1695 cm^−1^ remains unchanged, whereas the band at 1730 cm^−1^ exhibits a red‐shift to lower wavenumber, suggesting that water molecules could partially break the weak hydrogen bonds of COOH groups in PMAA and participate in the hydrogen bonding with C═O groups. From those points of view, the trace water absorbed from the ambient air functions as a structural component in the plastic instead of merely a plasticizer, which might contribute to excellent water resistance. In addition, the toughening effect on the mechanical performance with absorbed water might be attributed to the hierarchical hydrogen bond breaking during stretching to dissipate energy.

In addition to hydrogen bonds between water molecules and polymer chains, inter‐/intra‐chain hydrogen bonds are also responsible for the high performance and sustainability of the plastic. To figure out the hydrogen bonding structures, FTIR spectra of HPMC, PMAA, and HPMC_1_/PMAA_3_ in dry states were compared in Figure 4d, in which the band at ≈1700 cm^−1^ corresponds to *v*(C═O) of PMAA and the band at 1060 cm^−1^ is related to *v*(C─O─C) of HPMC. In the dry HPMC_1_/PMAA_3_ plastic, both *v*(C═O) and *v*(C─O─C) exhibit a higher wavenumber content increase, indicating that the hydrogen bonds in pure PMAA or HPMC could partially disassociate and transform into hydrogen bonding of COOH (PMAA)…O─C (HPMC). Furthermore, FTIR spectra of HPMC/PMAA plastics with varied compositions in the *v*(C═O) region have been collected to examine the effect of feeding ratio on the molecular interactions, as shown in Figure S19 (Supporting Information). With PMAA content decreasing, the intensity at ≈1695 cm^−1^ decreases whereas the intensity at ≈1730 cm^−1^ increases, indicating that the incorporation of HPMC to PMAA would partially break the strong dimeric hydrogen bonds in PMAA and free a part of COOH groups to participate in the hydrogen bonding with ether groups in HPMC. Herein, the well‐balanced hydrogen bonds of dimeric hydrogen bonding in PMAA and interchain acid‐ether hydrogen bonding of HPMC and PMAA might contribute to an optimized mechanical performance for HPMC_1_/PMAA_3_. To further evaluate the hydrogen bonding structures in the plastic, 2D correlation spectra (2Dcos),^[^ ^20^ ^]^ which would provide details on the internal interactions with the investigated system, were generated from the temperature‐dependent FTIR spectra of HPMC_1_/PMAA_3_ plastic from 30 to 180 °C (Figure 4e). Herein, splitting bands of 1743, 1726, 1693, and 1647 cm^−1^ related to C═O groups in PMAA and subtle bands at 1074 and 1024 cm^−1^ corresponding to C─O─C groups in HPMC have been recognized from the 2Dcos spectra (Figure 4f). Specifically, those bands from high to lower wavenumber could be tentatively assigned to *v*(C═O) (weakly hydrogen bonded with COOH), *v*(C═O) (hydrogen bonded with H_2_O), *v*(C═O) (dimeric hydrogen bond in PMAA), *δ*(O─H) (water), *v*(C─O─C) (free) and *v*(C─O─C) (hydrogen bonded with COOH), respectively, confirming the diverse hydrogen bonds in the plastic. For clarity, hydrogen bonding structures in the plastic are schematically illustrated in Figure 4g.

## 3 Conclusion

In this work, we develop a facile yet effective strategy to fabricate a new plastic with simultaneous sustainability and high performance. Our strategy involves in situ polymerization of MAA with the presence of HPMC in a quasi‐solvent‐free system, whose viscosity can be conveniently regulated by changing the HPMC/MAA ratio or adding a small amount of water, thus leading to the easy processing of the precursor mixture into diverse shapes via 3D printing, compression molding, etc. This approach requires no complicated purification or post‐treatment and allows the incorporation of various functional components into the plastic. Furthermore, the as‐obtained plastic can be reprocessed into other complex structures with hydrosetting. Moreover, the plastic can be recycled under mild conditions owing to its supramolecular nature. Under such circumstances, plastic is highly sustainable during production, processing, and recycling. Meanwhile, the plastic exhibits excellent mechanical properties with *E* up to 3.7 GPa and *σ*_b_ up to 150.2 MPa, superior to many commercially available plastics as well as other reported sustainable plastics. In addition, impressive thermal and solvent stability is observed for the plastic possibly due to the hierarchical hydrogen bonding interactions. This work provides a green manufacturing process to construct high‐performance and sustainable plastics toward actual load‐bearing scenarios.

## 4 Experimental Section

### Materials and Chemicals

HPMC was provided by Aladdin Reagents (viscosity of 3–6 mPa.s in 2 wt% aqueous solution at 20 °C). MAA was purchased from Sigma–Aldrich and purified by passing through an alumina column. 2‐hydroxy‐2‐methylpropiophenone (Irgacure 1173) was obtained from Aladdin Reagents. Curcumin was purchased from MACKLIN Chemical. Disperse Red 1 was purchased from J&K Chemical. All the reagents were used without further purification.

### Preparation of HPMC/PMAA Plastic

The HPMC/PMAA plastics were prepared by polymerizing MAA with the presence of HPMC and a small amount of water. Herein, the mixing and processing methods depend on the viscosity of the polymerization precursor, which was determined by MAA/HPMC weight ratio and the amount of water. The final polymerization process proceeded under 365 nm UV light for 1 h. The photoinitiator, Irgacure 1173, was fixed at 1 wt% of MAA. The resulting product was immersed in a large amount of water to remove unreacted residues. The plastic was finally obtained by air‐drying. When MAA/HPMC weight ratio and water content were low, the polymerization precursor was nearly solid and could be mixed by kneading and homogenized on a household noodle machine, allowing for hand molding into various shapes. With a high MAA/HPMC weight ratio and water content, the polymerization precursors were in viscous liquid states, which were mixed and homogenized using a mixer (Thinky ARE‐310 Planetary Centrifugal Mixer). The viscos precursors can be further processed with injection molding, compression molding, and 3D printing. The CDs were synthesized by hydrothermal method using citric acid and urea as raw materials.^[^ ^21^ ^]^ For plastic functionalization, CD and curcumin were introduced into the polymerization precursors simultaneously with HPMC and MAA mixing.

### Characterization

The transparency of the plastic (film thickness ≈300 µm) was examined on Lambda 950, PerkinElmer. Tensile tests of samples were conducted on a universal testing machine (Instron 5966 Dual Column Tabletop Testing System) at a stretching rate of 5 min^−1^. DMA analyses of the plastic were measured using a Solid analyzer (Model TA Instruments RSA‐G2). The RH was controlled by the temperature and humidity chamber (KMF 115, German binder). Temperature sweeps of DMA analysis were conducted on a Solid analyzer (Model TA Instruments RSA‐G2) at a heating rate of 5 °C min^−1^. Humidity sweep of DMA analysis was performed at 25 °C on a TA Q800 machine equipped with a humidity‐controlling accessory with a humidification rate RH 2% min^–1^. A cross‐section image of the plastics was taken on an SEM (Hitachi, Japan SU8230). 3D printing of the polymerization precursor was conducted on Regenovo 3D Bio‐Architect Sparrow. LF‐^1^H NMR measurements were performed by an NMR analyzer (VTMR20‐010V‐I, Suzhou Niumag Analytical Instrument Corporation, China) at room temperature. FTIR spectra were collected on a Nicolet iS50 (Thermo Fisher Scientific) spectrometer. Temperature‐dependent FTIR spectra of HPMC_1_/PMAA_3_ plastic were recorded in the transmission mode and collected continuously every 2 °C min^−1^ from 30 to 180 °C. 2Dcos analysis of the temperature‐dependent FTIR spectra was performed on the software 2D Shige ver. 1.3 (Shigeaki Morita, Kwansei–Gakuin University, Japan, 2004–2005). 2Dcos contour maps were plotted with Origin, where warm colors (red) indicate positive intensities, while cold ones (blue) indicate negative intensities.

## Conflict of Interest

The authors declare no conflict of interest.

## Supporting information

Supporting Information

## Data Availability

The data that support the findings of this study are available from the corresponding author upon reasonable request.
