# Supplementary material for: Sustainable Plastics with High Performance and Convenient Processibility
Source: Adv Sci (Weinh). 2024 Jul 19;11(35):2405301. doi: 10.1002/advs.202405301 (PMC11529043; doi:10.1002/advs.202405301)
Supplement: Supplementary file 1 — Supporting Information [file ADVS-11-2405301-s001.docx]

Supporting Information

Sustainable Plastics with High Performance and Convenient Processibility

Guogang Xu, Lei Hou,* Peiyi Wu*

| 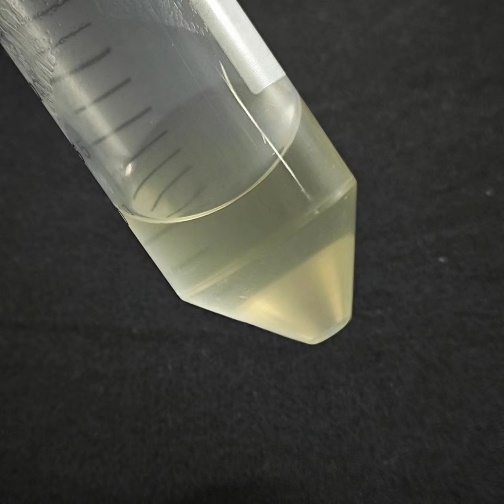 |
| --- |
| **Figure S1.** Photograph of the uniform mixture of HPMC and MAA in the weight ratio of 1:3 with 10 wt% water content. |

| **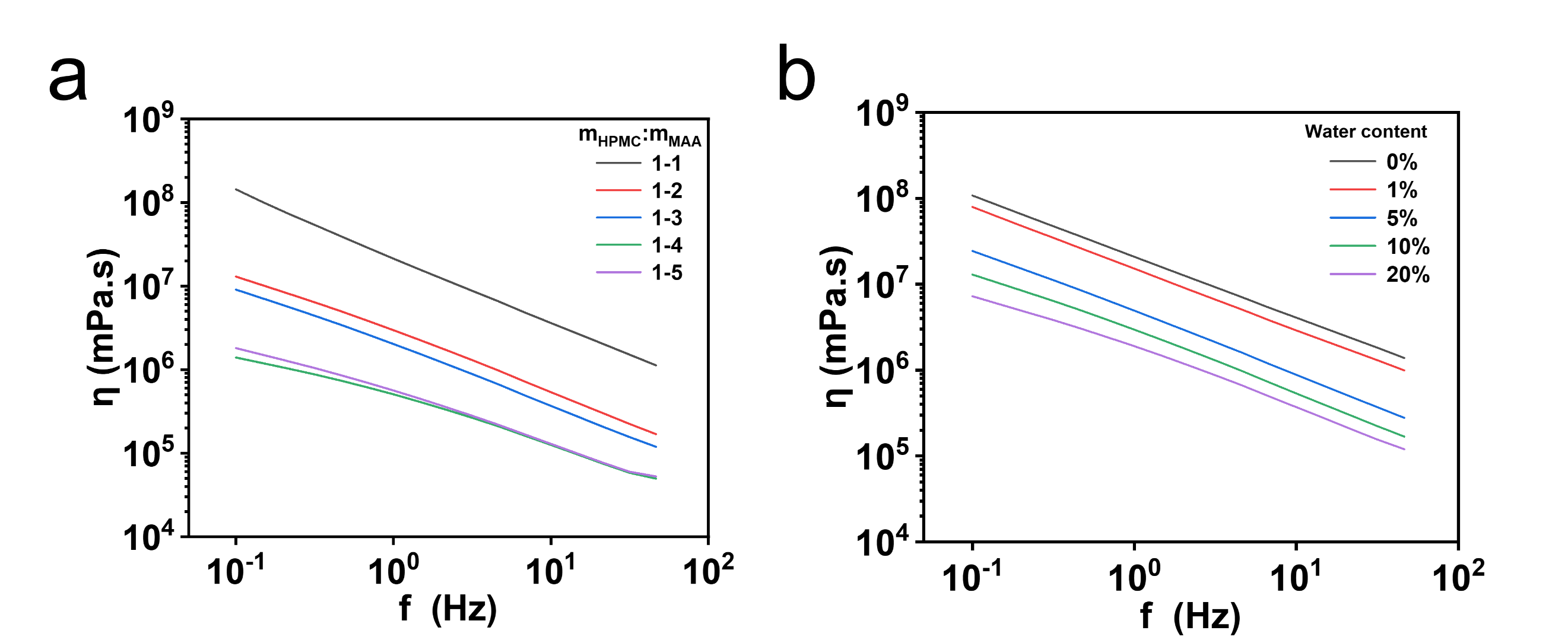** |
| --- |
| **Figure S2.** Viscosity of the polymerization precures of different HPMC/MAA ratio with 10 wt% water (a) and varied water content with m(HPMC): m(MAA)= 1: 3 (b). |

| **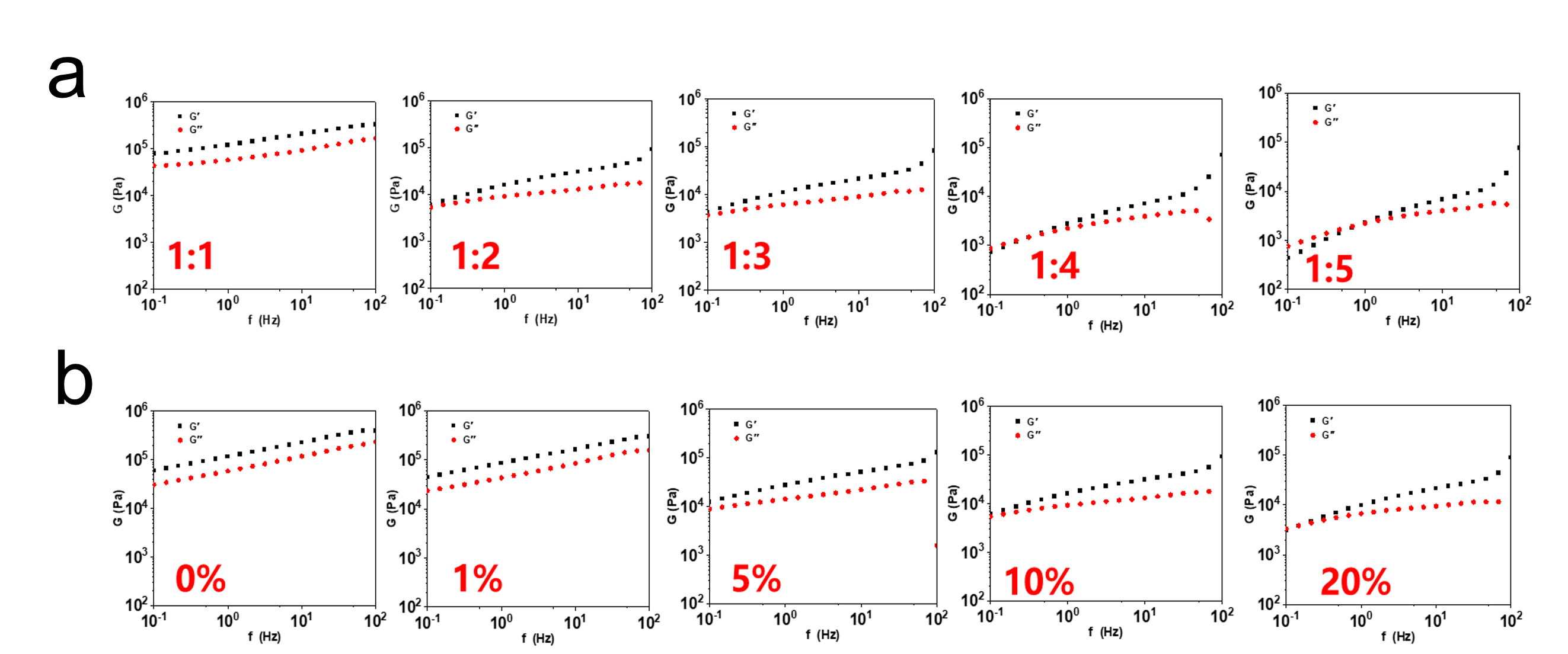** |
| --- |
| **Figure S3.** Rheological curves of the polymerization precursors of different HPMC/MAA ratio with 10 wt% water (a) and varied water content with m(HPMC): m(MAA) = 1: 3 (b). |

| **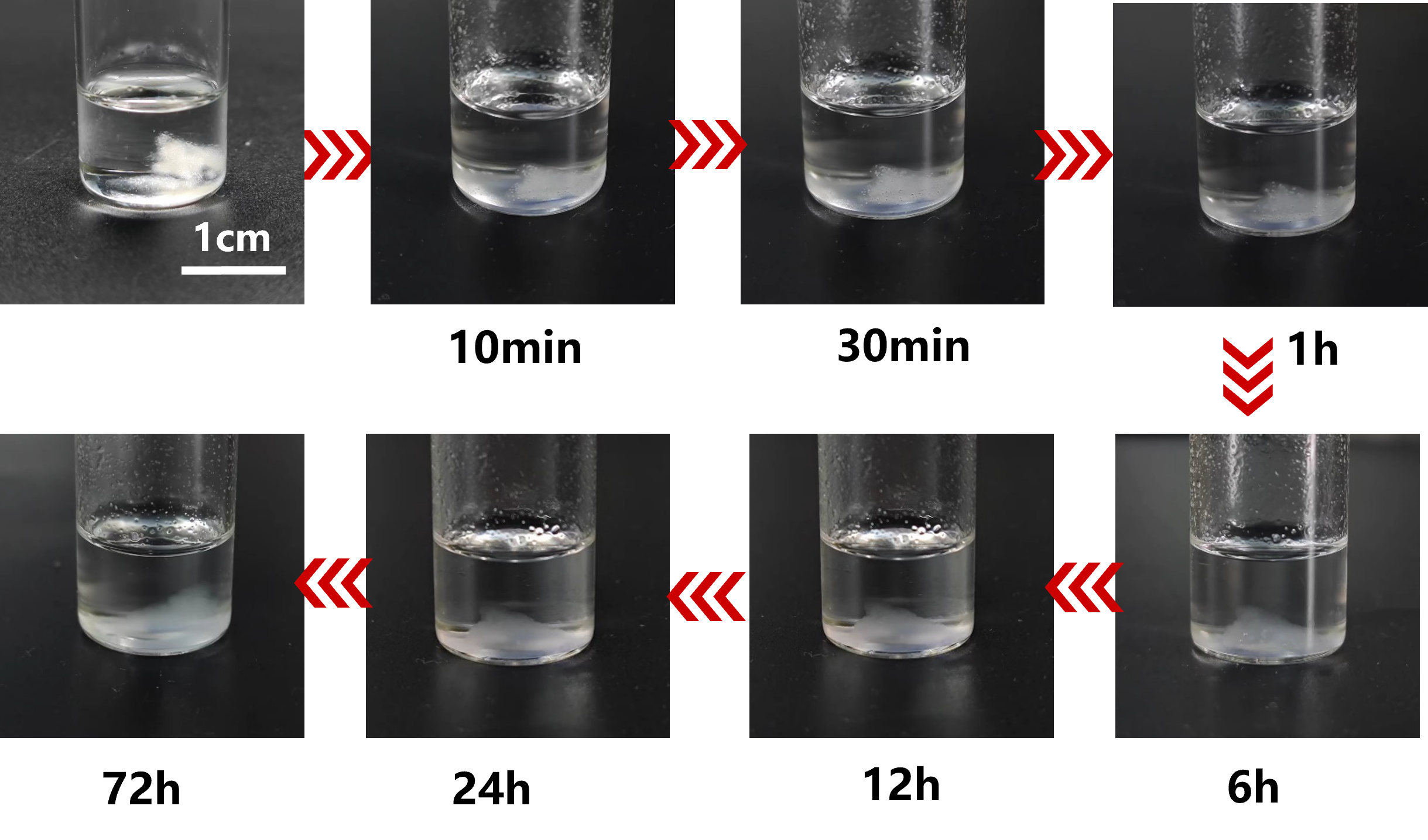** |
| --- |
| **Figure S4.** Photographs of HPMC swollen in MAA. |

| 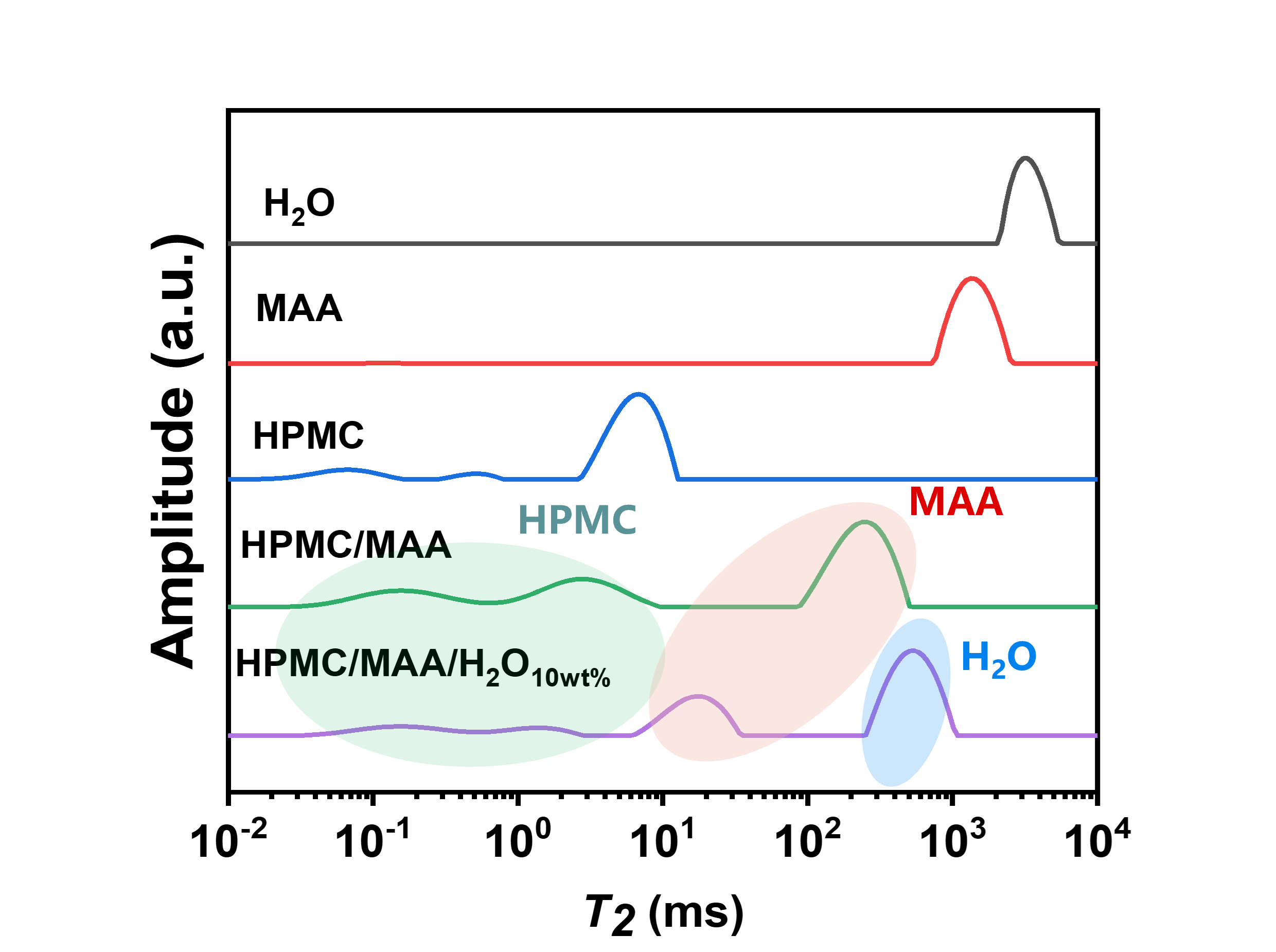 |
| --- |
| **Figure S5.** LF-^1^H NMR spectra of H_2_O, MAA, HPMC, HPMC_1_/MAA_3_, and HPMC_1_/MAA_3_/H_2_O_10 wt%_. |

| **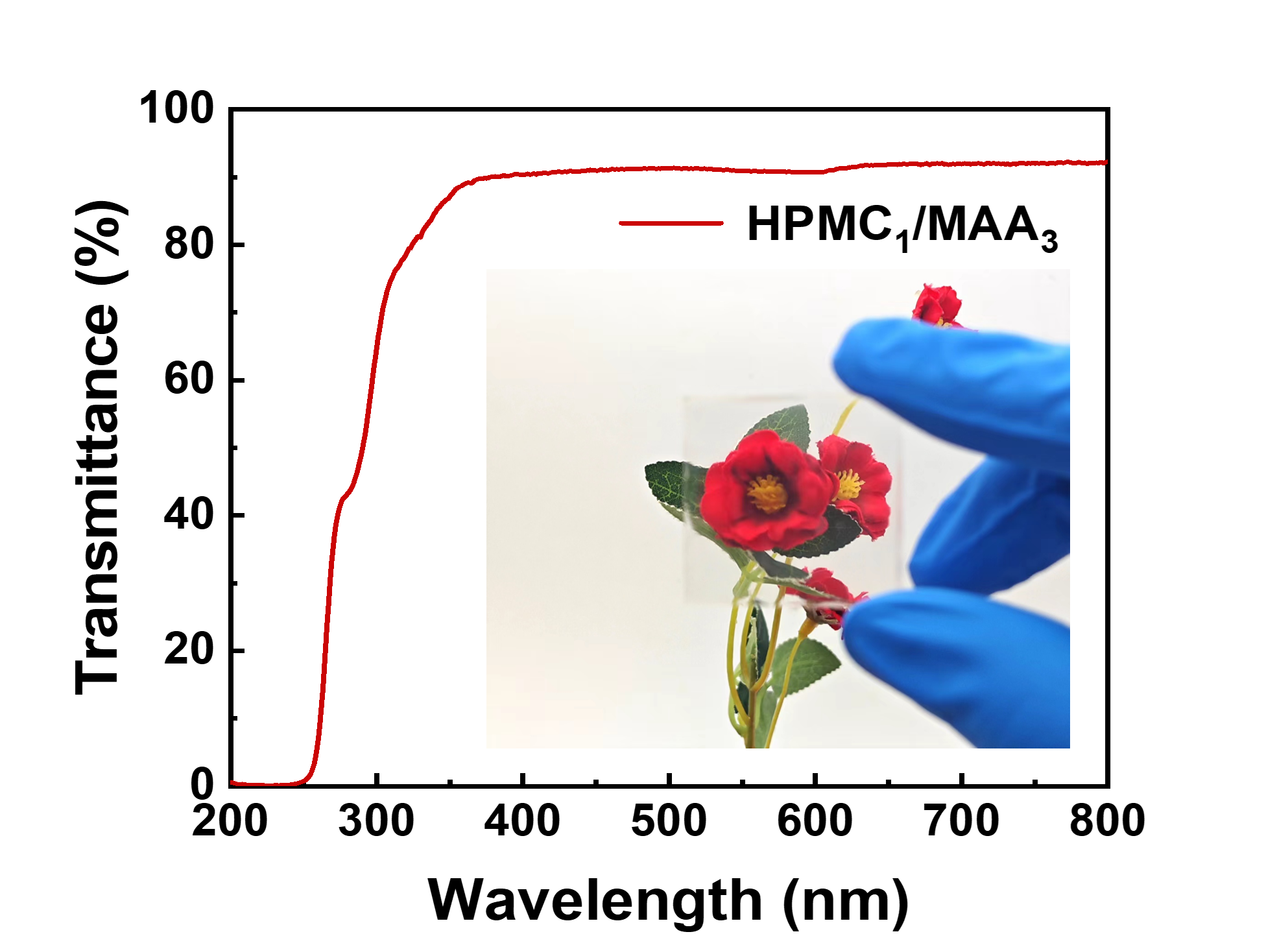** |
| --- |
| **Figure S6.** UV-vis spectrum and photograph of the HPMC_1_/PMAA_3_ plastic (film thickness ≈500 µm). |

| **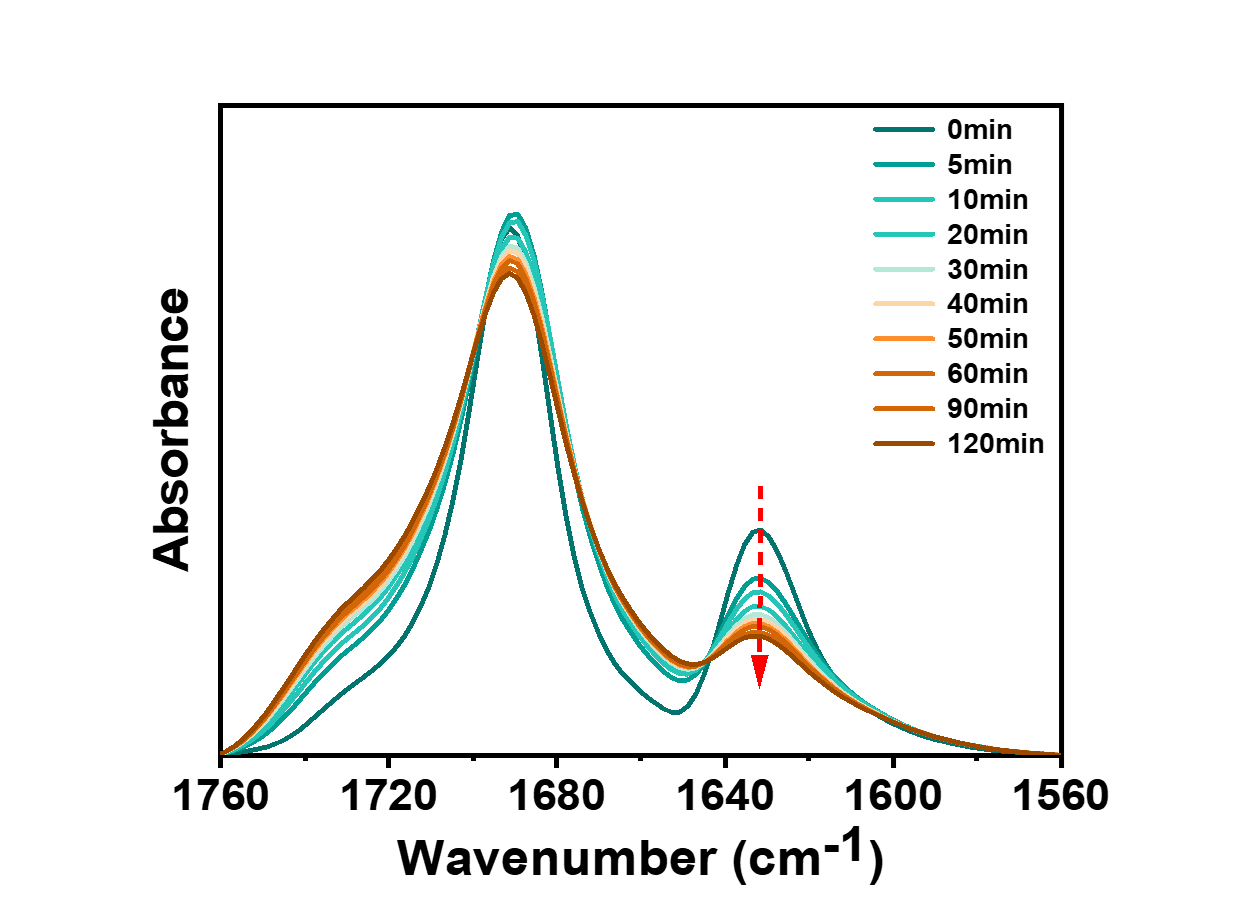** |
| --- |
| **Figure S7.** Time-dependent FTIR spectra of the HPMC_1_/MAA_3_ during polymerization. |

| 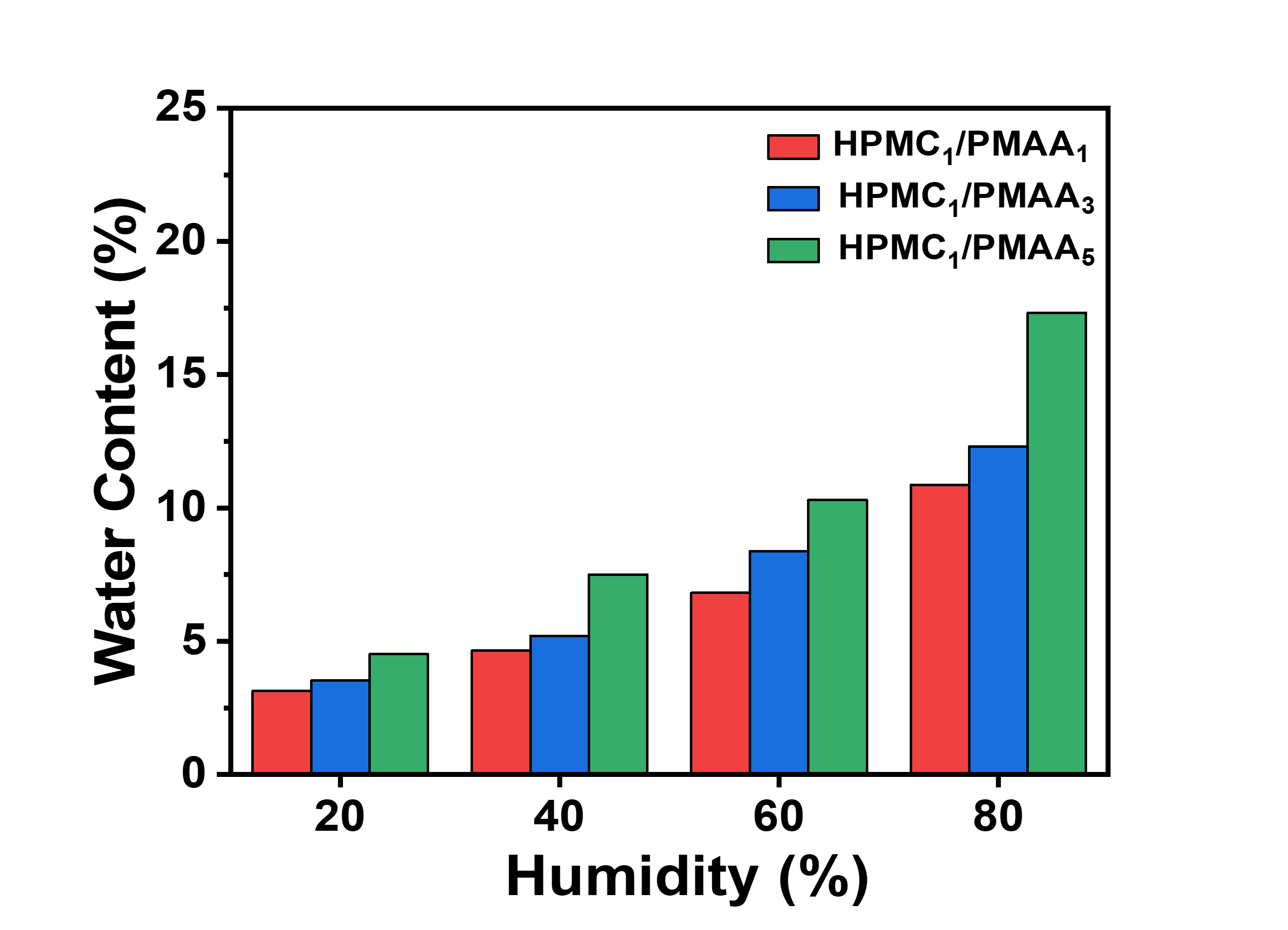 |
| --- |
| **Figure S8.** Water content of HPMC/PMAA plastics equilibrated at different RH. |

| **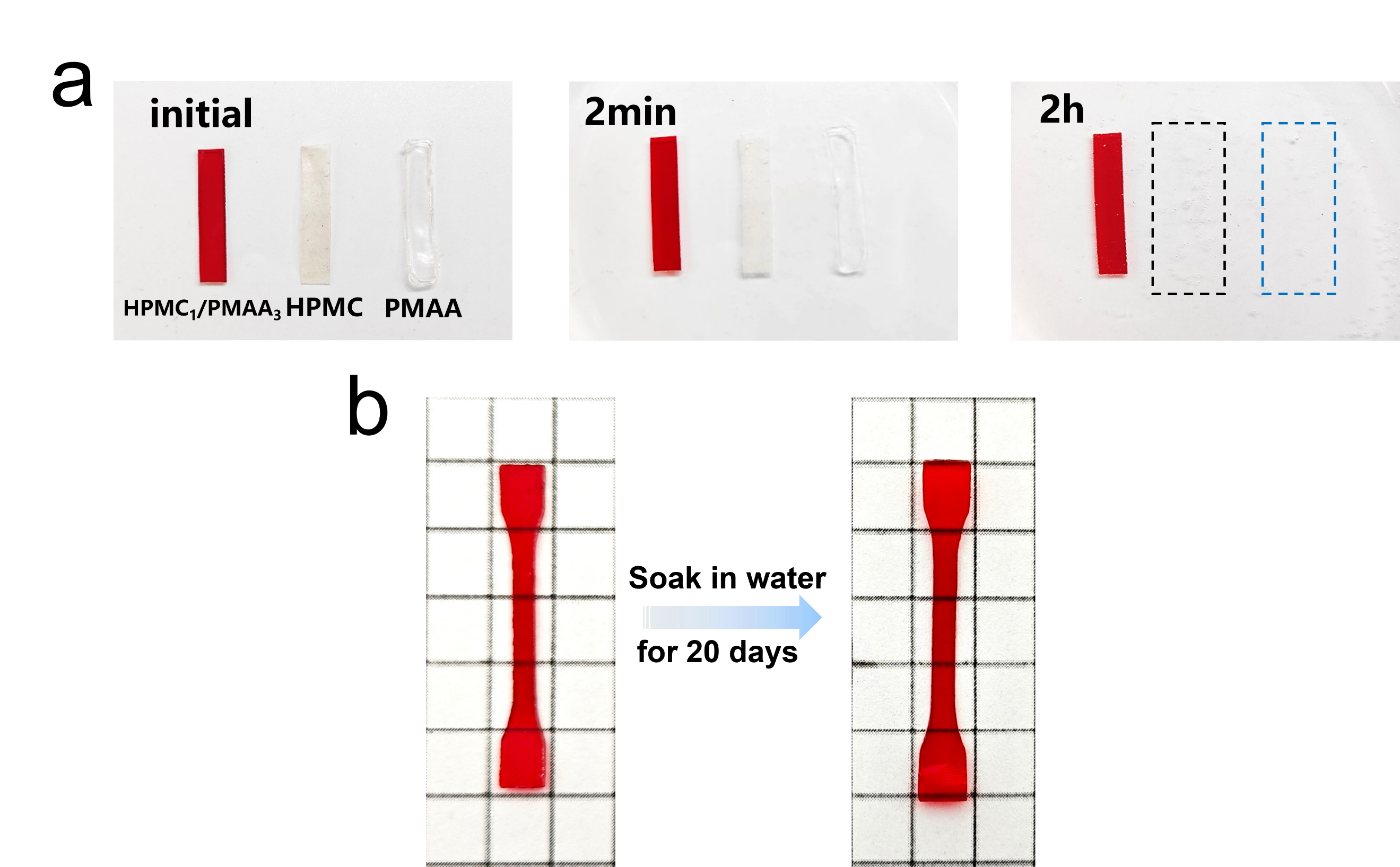** |
| --- |
| **Figure S9.** Photographs of the HPMC_1_/PMAA_3_ plastic, HPMC, PMAA after soaking in water (a) and the HPMC_1_/PMAA_3_ plastic soaked in water for 20 days (b). |

| 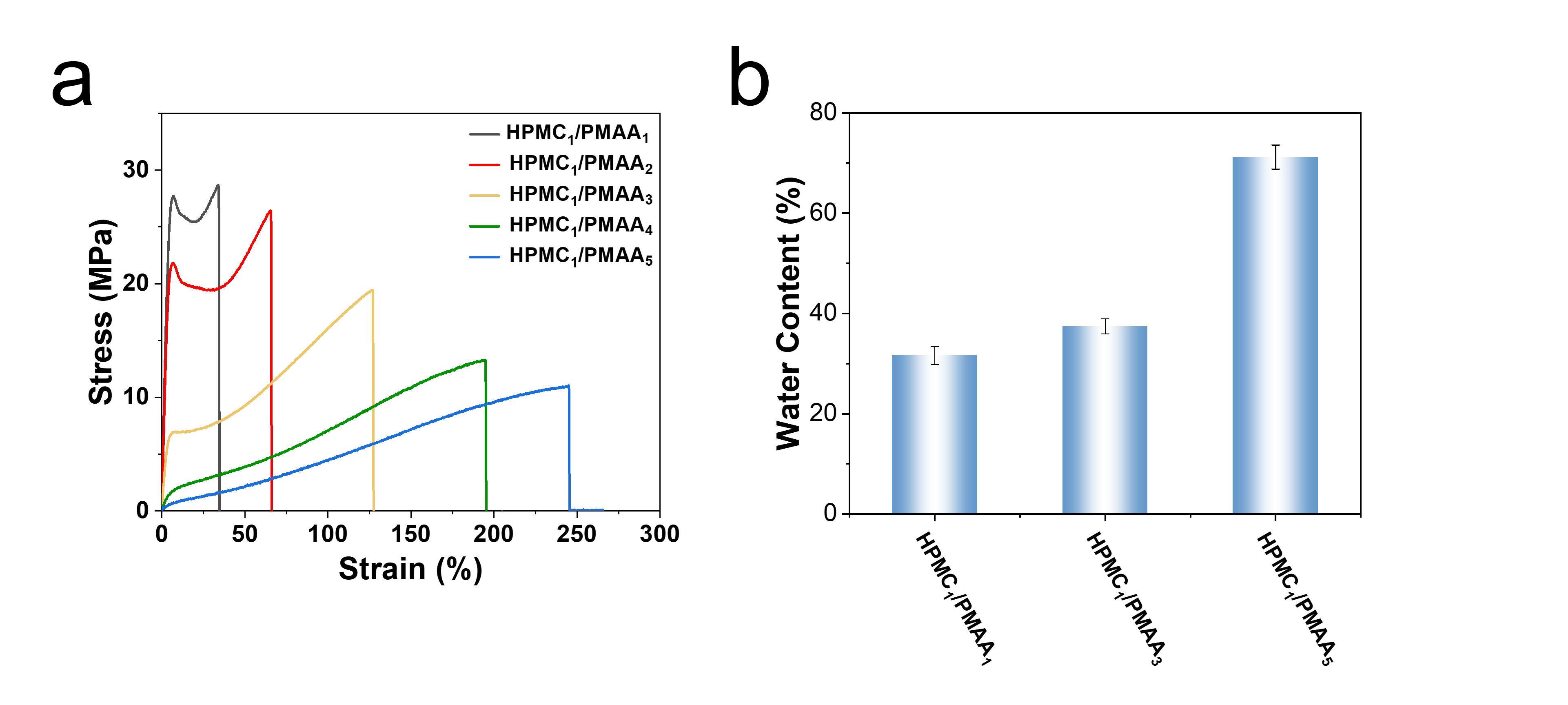 |
| --- |
| **Figure S10.** Tensile stress-strain curves (a) and water contents (b) of HPMC/PMAA hydrogels. |
|  |
| 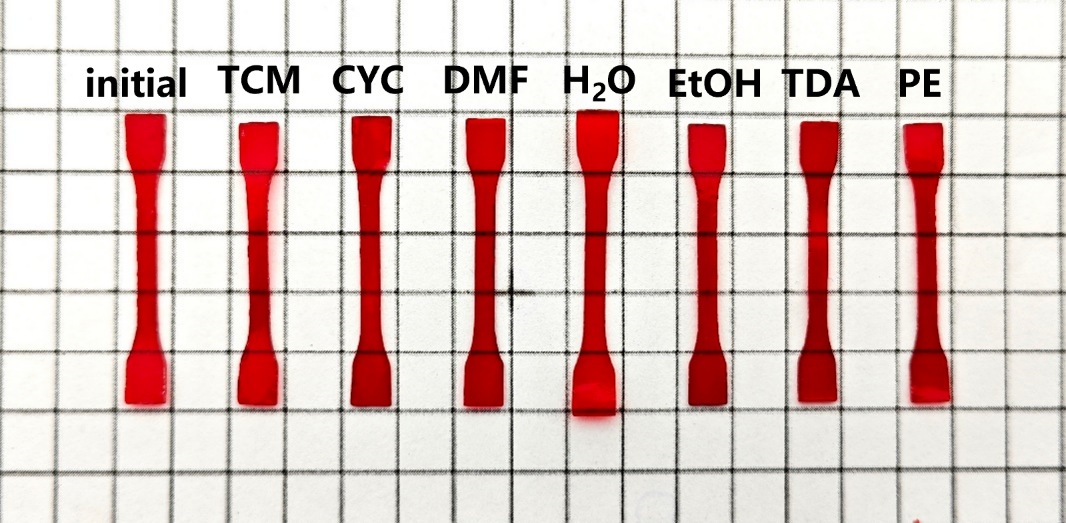 |
| **Figure S11.** The stability of HPMC/PMAA plastic in various solvents after 20 days (TCM: trichloromethane, CYC: cyclohexanone, EtOH: ethyl alcohol, DMF: *N,N*-Dimethylformamide, TDA: toluenediamine, PE: petroleum ether). |

| 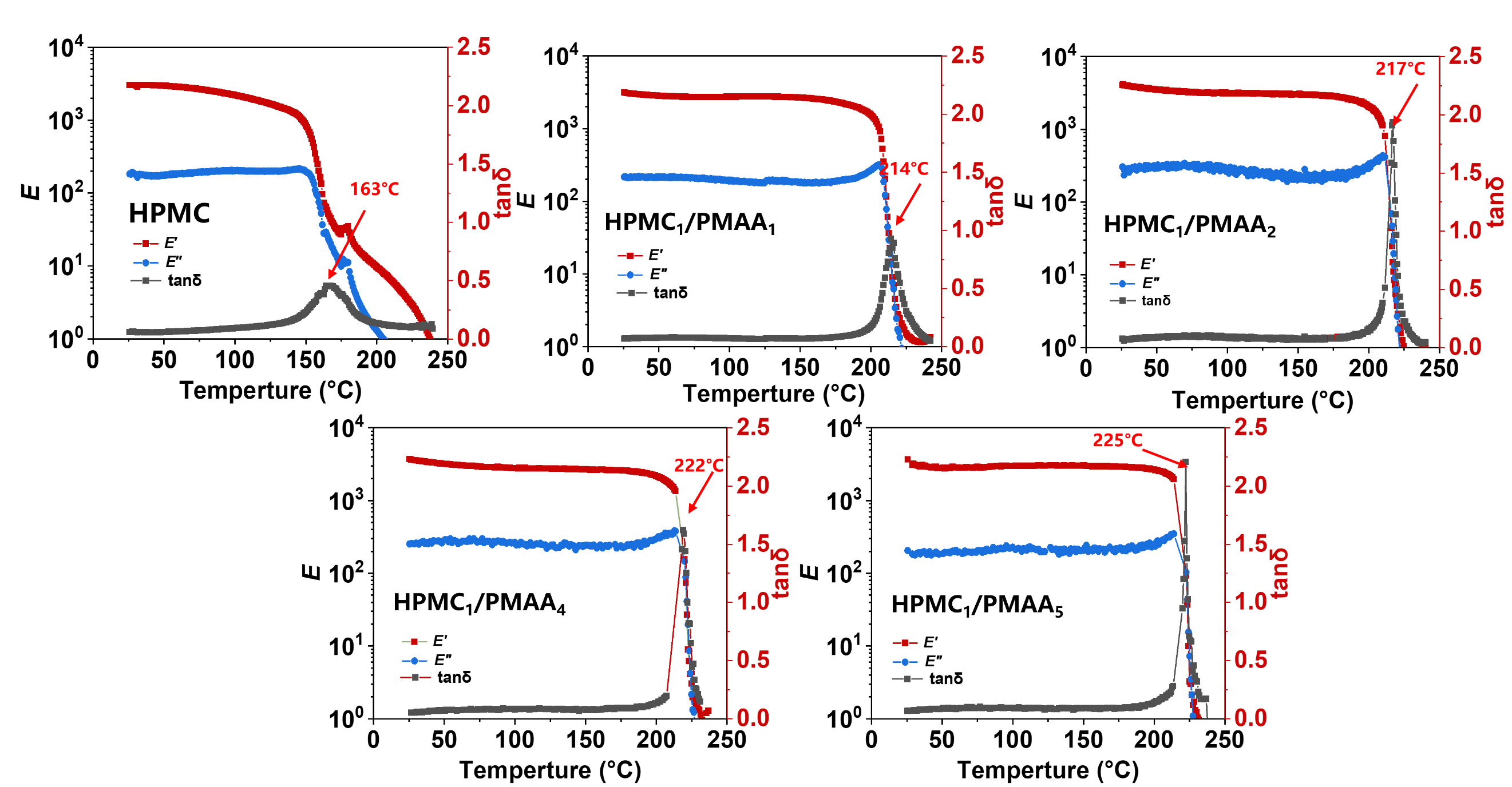 |
| --- |
| **Figure S12.** DMA curves of temperature sweep of HPMC/PMAA with different compositions. |

| 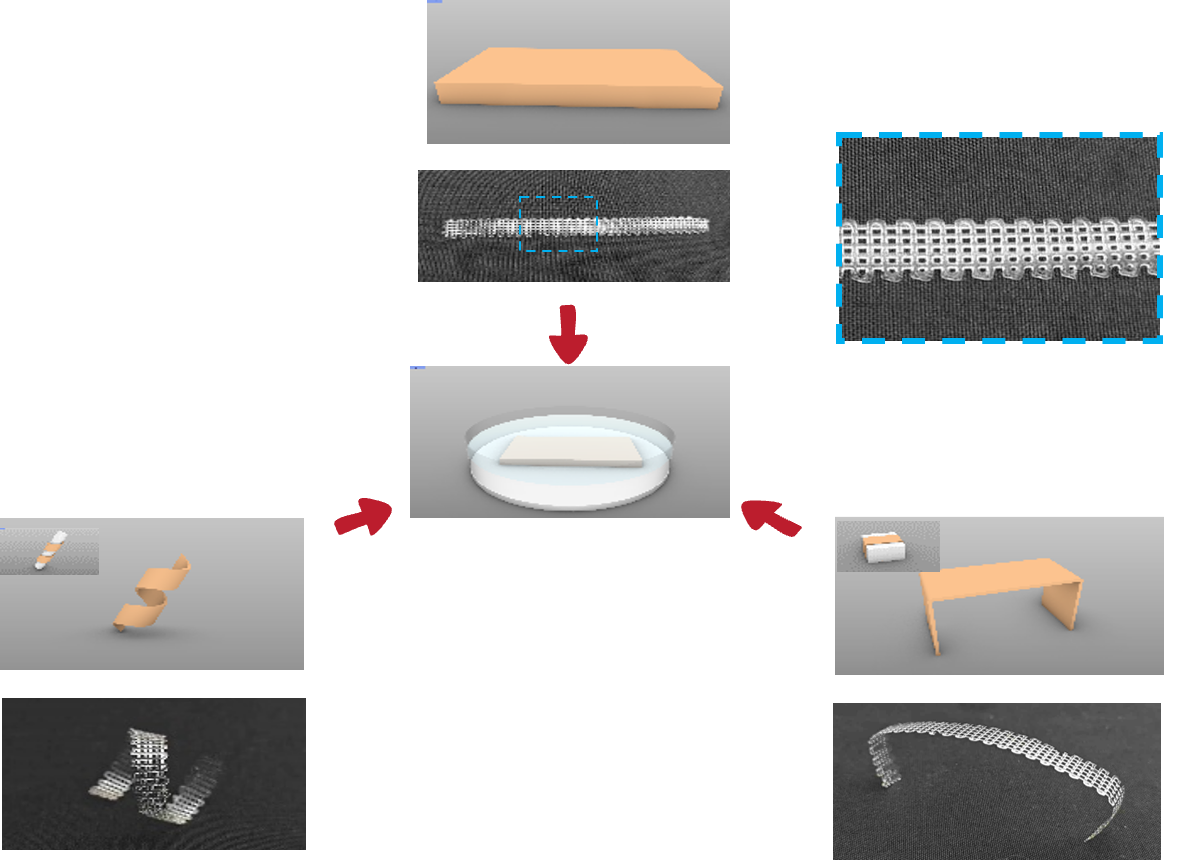 |
| --- |
| **Figure S13.** Schematic and Experimental demonstration of processing HPMC/PMAA plastic with sustainable hydrosetting method. |

| 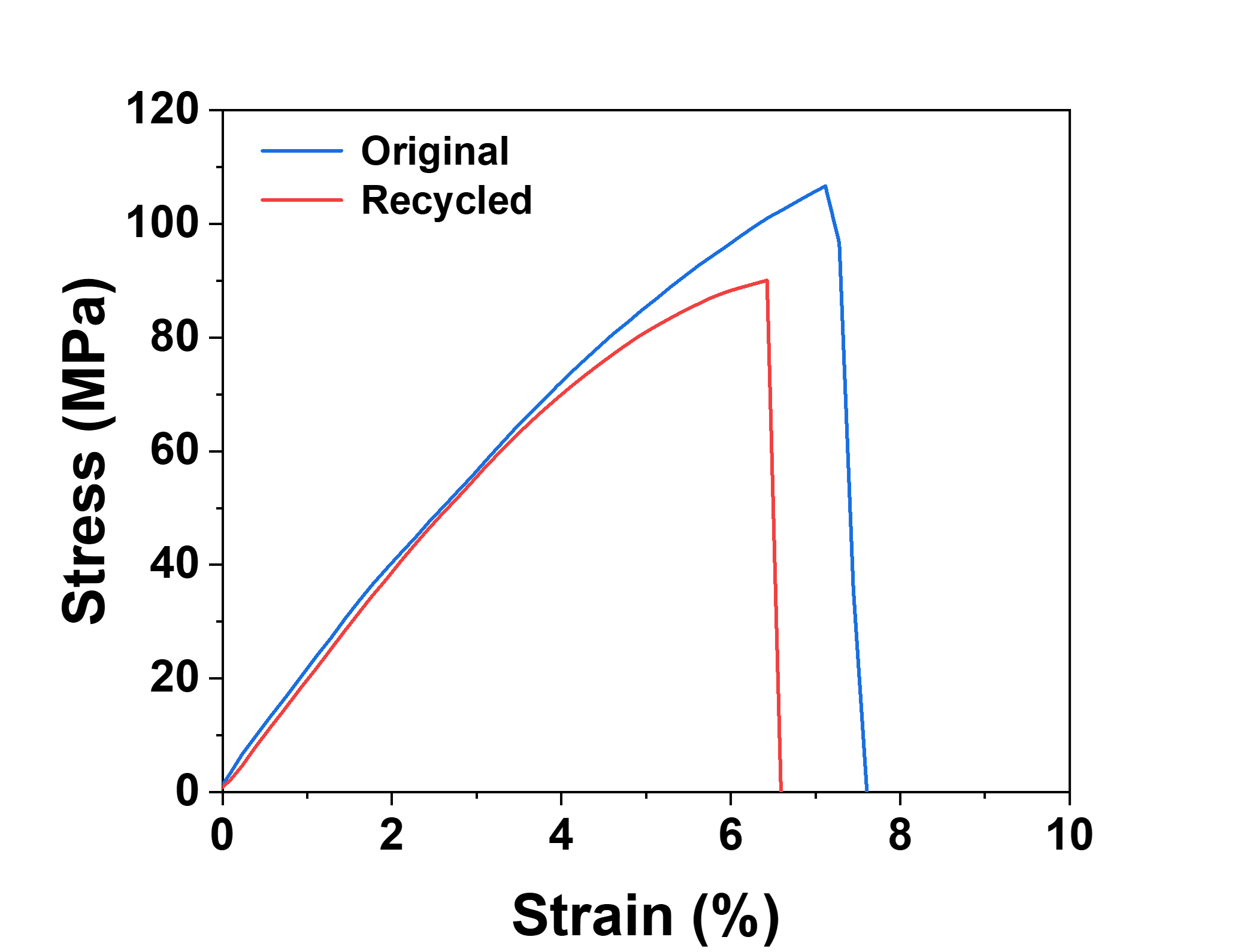 |
| --- |
| **Figure S14.** Tensile stress-strain curves of the original and recycled HPMC/PMAA plastics equilibrated under 50% RH. |

| **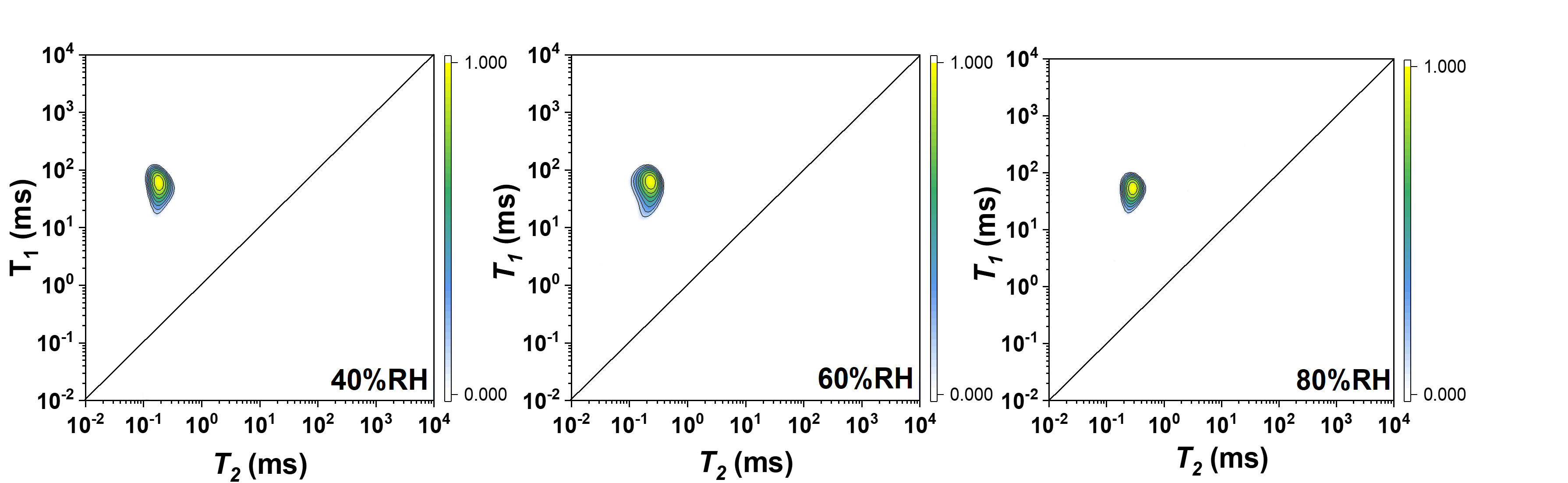** |
| --- |
| **Figure S15.** 2D LF-^1^H NMR spectra of the HPMC_1_/PMAA_3_ plastic equilibrated under different RH. |

| 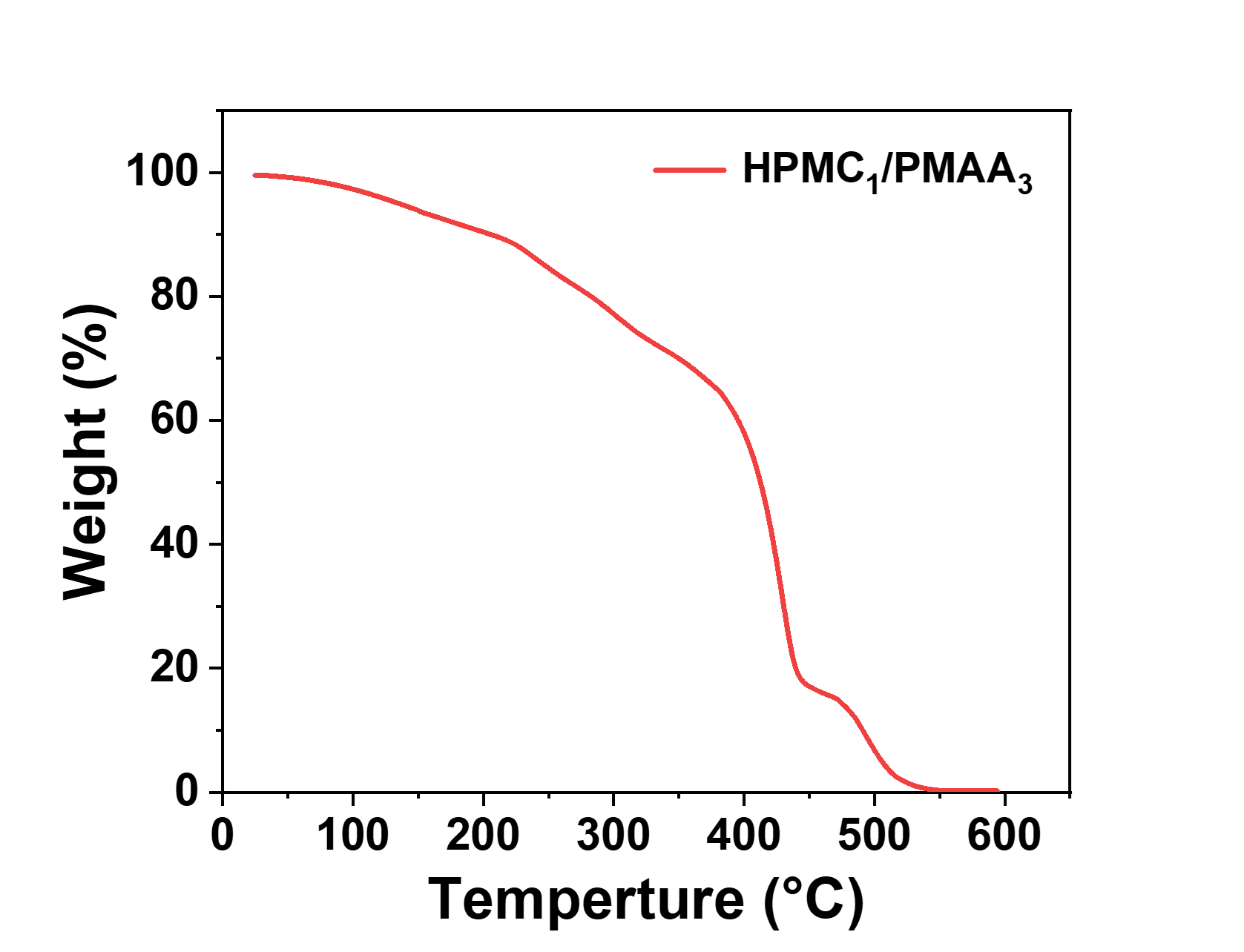 |
| --- |
| **Figure S16.** TGA curve of HPMC_1_/PMAA_3_ in air_._ Heating rate: 10 ^o^C/min. |

| **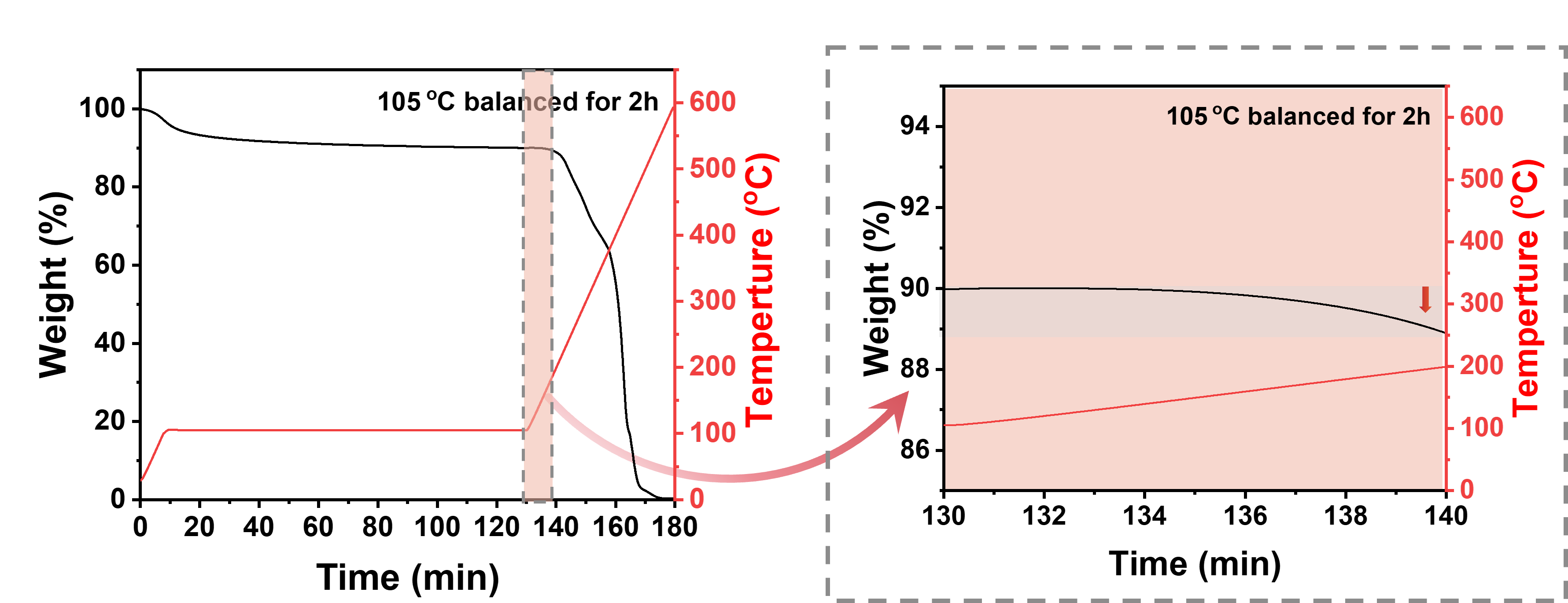** |
| --- |
| **Figure S17.** TGA curve of HPMC_1_/PMAA_3_ equilibrated at 105 ° C for 2 h and dried to constant weight in air (Heating rate: 10 ^o^C/min). |

| 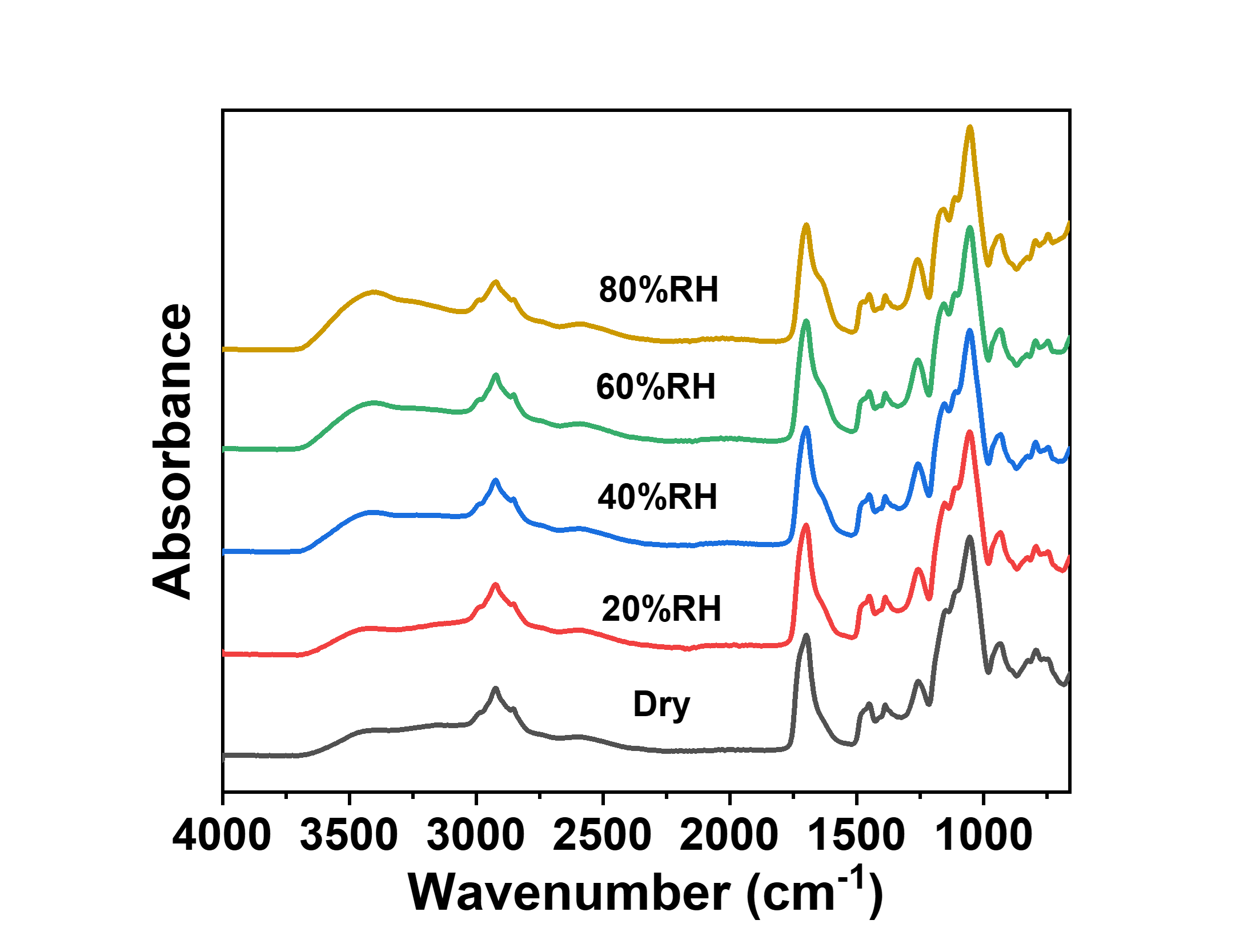 |
| --- |
| **Figure S18.** FTIR spectra of HPMC_1_/PMAA_3_ equilibrated under different RH. |

| **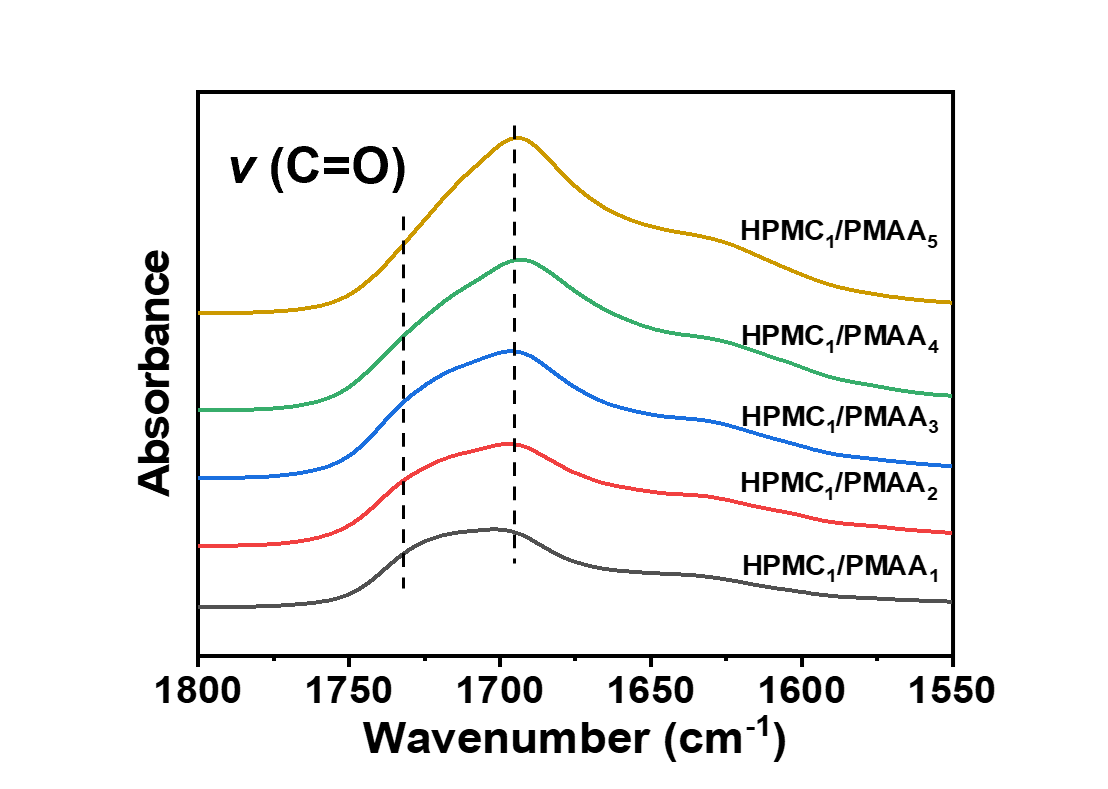** |
| --- |
| **Figure S19.** FTIR spectra of HPMC/PMAA plastics with different feeding ratios. |
